# *pyXPCSviewer*: an open-source interactive tool for X-ray photon correlation spectroscopy visualization and analysis

**DOI:** 10.1107/S1600577522004830

**Published:** 2022-06-07

**Authors:** Miaoqi Chu, Jeffrey Li, Qingteng Zhang, Zhang Jiang, Eric M. Dufresne, Alec Sandy, Suresh Narayanan, Nicholas Schwarz

**Affiliations:** a X-ray Science Division, Argonne National Laboratory, 9700 South Cass Avenue, Argonne, IL 60439, USA

**Keywords:** X-ray photon correlation spectroscopy, synchrotron, visualization, GUI, Python

## Abstract

*pyXPCSviewer*, a Python-based graphical user interface that is deployed at beamline 8-ID-I of the Advanced Photon Source for interactive visualization of XPCS results, is introduced. *pyXPCSviewer* parses rich X-ray photon correlation spectroscopy (XPCS) results into independent *PyQt* widgets that are both interactive and easy to maintain. *pyXPCSviewer* is open-source and is open to customization by the XPCS community for ingestion of diversified data structures and inclusion of novel XPCS techniques, both of which are growing demands particularly with the dawn of near-diffraction-limited synchrotron sources and their dedicated XPCS beamlines.

## Abstract 2

The Python-based graphical user interface *pyXPCSviewer* that is deployed at beamline 8-ID-I of the Advanced Photon Source for interactive visualization of X-ray photon correlation spectroscopy results is introduced.

## 1. Introduction

X-ray photon correlation spectroscopy (XPCS), whose underlying principles are derived from photon correlation spectroscopy (PCS), is a coherent X-ray scattering technique enabled by high-brilliance undulator sources as it relies on the spatial coherence of the X-ray beam to generate far-field interference patterns (‘speckles’) in the scattered beam intensity. In addition to the structural information *I*(*Q*) encoded in X-ray scattering (*e.g.* small-angle X-ray scattering, SAXS), where *I* is the scattering intensity and *Q* is the momentum transfer, XPCS records *I*(*Q*, *t*) of intensity speckles over time *t*. As a result, XPCS provides direct measurement of system dynamics via the temporal intensity correlation function, *e.g.* *g*_2_(τ, *Q*) = 〈*I*(*t*, *Q*)*I*(*t* + τ)〉_*t*_/〈*I* ^2^(*t*, *Q*)〉_*t*_. For the simplest XPCS measurements, the beam on the sample is fixed and the statistics of *g*_2_ can be greatly enhanced by binning detector pixels over *Q* and averaging over *t* assuming that the dynamics are stationary during intensity collection (Dierker *et al.*, 1995 ▸). For more details about XPCS theories and experimental setup, readers can refer to Grübel *et al.* (2008 ▸) and Shpyrko (2014 ▸).

The versatility of XPCS, combined with full compatibility with *in situ* sample environments and wide temporo-spatial coverage, has led to unique insight into the collective atomic motion in metallic glasses (Evenson *et al.*, 2015 ▸; Giordano & Ruta, 2016 ▸) and supercooled liquid alloys (Ruta *et al.*, 2020 ▸), arrested dynamics of nanoparticles in polymer matrices (Dierker *et al.*, 1995 ▸; Senses *et al.*, 2017 ▸; Yavitt *et al.*, 2021 ▸), liquid–liquid phase separation in protein (Begam *et al.*, 2021 ▸) and micelle suspensions (Sheyfer *et al.*, 2020 ▸), and the mesoscopic structure dynamics in both abrasive manufacturing such as ion-beam patterning (Myint *et al.*, 2021 ▸) and additive manufacturing such as 3-D printing (Lin *et al.*, 2021 ▸), where the results common to all these XPCS applications are intensity correlation functions in the form of multi-dimensional spectra over time and reciprocal space. However, generating and interpreting XPCS results can be very challenging due to the multi-dimensional and statistical nature of the data that require efficient reduction and sophisticated visualization tool suites. This challenge will be further heightened by more sophisticated XPCS analysis including the higher-order (Perakis *et al.*, 2017 ▸; Dallari *et al.*, 2020 ▸) and higher-dimensional (Sutton *et al.*, 2021 ▸) correlation functions enabled by the orders-of-magnitude increases of coherent flux from emerging near-diffraction-limited synchrotron sources around the world such as APS-U (Shi *et al.*, 2017 ▸), ESRF-EBS (Chenevier & Joly, 2018 ▸), PETRA-IV (Schroer *et al.*, 2018 ▸) and MAX-IV (Martensson & Eriksson, 2018 ▸). A suite of expandable, customizable and user-friendly XPCS software tools is therefore essential for advancing the scientific impact of XPCS.

A Matlab-based graphical user interface (GUI) for visualizing XPCS results, *XPCSGUI* (Sikorski *et al.*, 2011 ▸), is currently deployed at APS 8-ID. However, due to the limitation of the Matlab proprietary platform, a growing amount of software at synchrotron X-ray facilities is being developed using open-source tools (Rio & Rebuffi, 2019 ▸), with the existing Matlab GUIs either transitioning to Python (Rogers *et al.*, 2019 ▸) or having supplementary Python versions (Lou *et al.*, 2021 ▸; Schirmer & Althaus, 2020 ▸; Zhang *et al.*, 2014 ▸). A Python-based GUI for XPCS will not only benefit significantly from the foundation already laid by the community but will, we hope, also become an organic component for the ongoing development of an open-source Python-based ecosystem at multiple synchrotron facilities, including the Python-based beamline control system (Bluesky) at NSLS-II (Arkilic *et al.*, 2017 ▸) and the Data Management workflow at APS (Veseli *et al.*, 2018 ▸).

## 2. Package structure

*pyXPCSviewer* follows the standard Model-View-Controller (MVC) pattern (Gamma *et al.*, 1995 ▸) for quick development and easy maintenance. The GUI takes users’ controls (mouse click/movement) and inputs and displays the images and plots, while *pyXPCSviewer*’s kernel (Model) fetches and processes the data for display.

*pyXPCSviewer* is written in Python so it can run easily on major platforms, including Windows, MacOS and Linux. The GUI functions are enabled by a combination of *PyQt* (Riverbank Computing, 2022 ▸), *PyQtGraph* (Campagnola, 2022 ▸) and *Matplotlib* (Hunter, 2007 ▸) that provide performance, interactivity and ready customization. The compute-intensive operations of *pyXPCSviewer* are performed with *NumPy* (Harris *et al.*, 2020 ▸) and *SciPy* (Virtanen *et al.*, 2020 ▸). The code and installation guide are available on GitHub (Chu, 2021 ▸)

**Figure 1:**
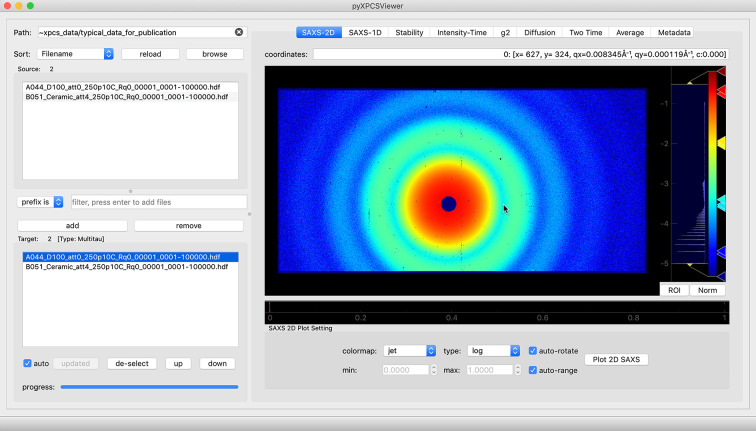
Layout and modules for *pyXPCSviewer*. In the left panel, users can set or reload the working directory. The data files will be displayed in the source box with the option to be sorted and filtered. Files can be added, removed and sorted in the target box that lists the files for visualization. The right panel contains the visualization modules, organized in tabs — a 2-D scattering pattern is displayed in this figure.

Following standard practices optimized over many years of operations at 8-ID-I at the APS, the workflow of *pyXPCSviewer* is divided into the following six groups: (i) dataset selection and loading, (ii) inspection of scattering patterns, (iii) assessment of sample beam damage and beamline stability, (iv) visualization and modeling of multi-tau correlations, (v) visualization of two-time correlations, and (vi) helper functions. The functions in each group are organized as separate tabs in the GUI, as shown in Fig. 1 ▸, so that the information is centrally organized. Comprehensive help also accompanies each of the workflow steps.

### 2.1. Dataset loading

Currently, *pyXPCSviewer* supports the APS Data Exchange format based on HDF5 file structures (De Carlo *et al.*, 2014 ▸). The files are generated from XPCS-Eigen (Khan *et al.*, 2018 ▸), which computes the correlation of the raw data and combines all the metadata (such as X-ray energy, *Q*-map definition and beam position) as one file. Other data formats can also be loaded using an adaptor layer to map the data to the format used by *pyXPCSviewer*.

In the GUI shown in Fig. 1 ▸, users can specify the working directory interactively via the load button or as a command line argument when launching the viewer. The example data set in this manuscript was captured using XSPA-500k, a 1024 × 512 pixel array detector featuring gapless field-of-view and a maximum continuous frame rate of 52 kHz (Nakaye *et al.*, 2021 ▸). After a valid working directory is set, the source panel lists the available files. To locate and select datasets quickly, users can sort the source files using the filename, the scan index (the first four digits of the filename at the APS) and time of creation. In addition, two filter types are included to narrow down the possibilities, namely a prefix filter and a substring filter. Once added to the target box, the datasets are loaded into the computer’s RAM to accelerate subsequent operations. *pyXPCSviewer* supports loading multiple datasets allowing users to overlay the correlation curves to compare the differences in detail. This is only possible if the detector dimensions match and the *Q*-partition maps are the same.

### 2.2. Inspection of scattering patterns

The time-average scattering pattern from an XPCS dataset is obtained by averaging all frames in the time series. It contains information about the structure of the sample. *pyXPCSviewer* uses the ImageView module of *PyQtGraph* to display the 2-D scattering patterns. As shown in Fig. 1 ▸, it provides customizable colormaps, easy control over the data range and quick response during zoom in/out. By examining the displayed scattering pattern, many anomalies can be easily spotted, such as no beam, scattering streaks (parasitic scattering from slits or sample holders) or a misaligned beamstop. Such datasets can be excluded from further analysis to avoid artifacts in the correlation results or users can define a mask that excludes the abnormal regions.

**Figure 2:**
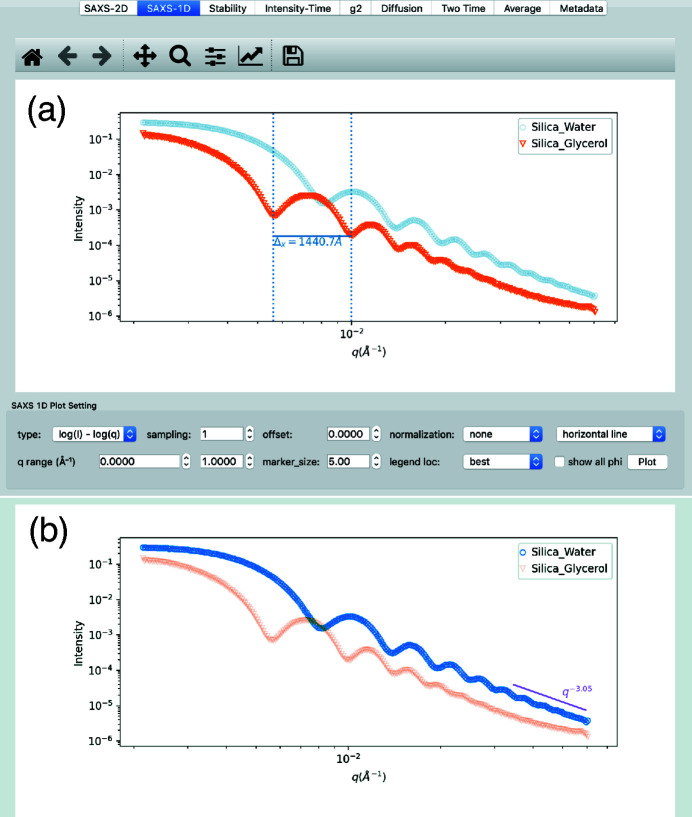
1-D small-angle scattering visualization. Users can draw horizontal lines (vertical dashed lines help users locate peaks/valleys) to estimate the structure size as shown in (*a*) or draw slopes to estimate the power-law decay constant as shown in (*b*).

In the small-angle geometry, if the sample is isotropic, it is convenient to perform azimuthal averaging of the scattering signal. Once collapsed to 1-D *I*(*Q*), users can change axis types (log or linear) and apply a normalization method if needed to enhance features or contrast of the profile. *pyXPCSviewer* provides two drawing methods as shown in Fig. 2 ▸ that allow users to draw lines interactively with the mouse. The horizontal line method allows users to draw horizontal lines between peaks/valleys to estimate the particle’s size, that is calculated as Δ_*x*_ = 2π/Δ_*Q*_, where Δ_*Q*_ is the length of the horizontal line. The other method is slope drawing that allows users to estimate the power-law parameter of *a* in the asymptotic relationship *I* ≃ *Q* ^*a*^. More advanced modeling of SAXS data is not provided but users can export the data to a dedicated SAXS package (Ilavsky & Jemian, 2009 ▸; Petoukhov *et al.*, 2012 ▸) for in-depth analysis.

### 2.3. Sample and beamline stability

XPCS is a technique that probes the dynamics of materials by correlating the scattering fluctuations. In order to investigate the inherent properties of the dynamic material system, it is necessary to exclude X-ray introduced artifacts (also known as X-ray beam damage) or fluctuations from the beamline. *pyXPCSviewer* enables quick inspection of the data so users can optimize data collection protocols at the beamline to identify and eliminate such artifacts.

**Figure 3:**
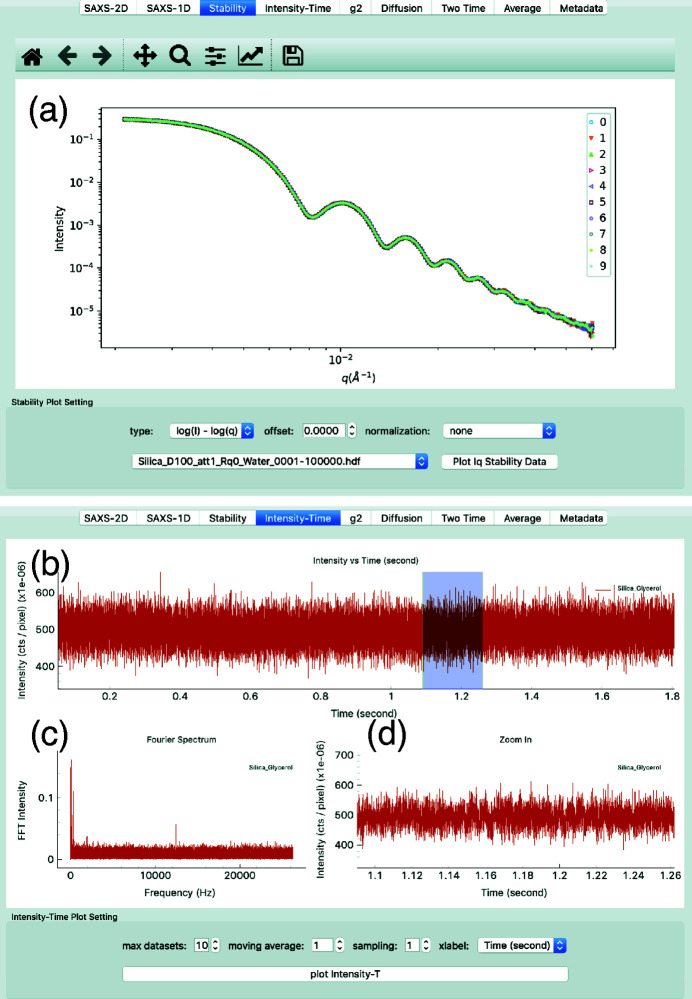
(*a*) Stability plot showing the SAXS 1-D *I*(*Q*) curves generated from segments of the time series. In this example, the ten curves match indicating that the sample is stable during the measurement. (*b*) The intensity versus time during the XPCS measurement. (*c*) Fourier component analysis of (*b*). (*d*) Zoom-in of the region highlighted by the light blue rectangle in (*b*).

The sample stability is characterized by dividing a time series into several continuous segments and computing the 1-D SAXS curve, *I*(*Q*), in each segment, as shown in Fig. 3 ▸(*a*). The curves should overlap with each other if the measurement does not suffer from artifacts. If the curves are different, users might have to attenuate the beam to reduce the radiation dose on the sample or use a different kind of sample. Inspection of the SAXS data for radiation-induced effects will become particularly critical with the marked increase of coherent flux from the next generation of X-ray sources.

We also need to make sure that the beamline is stable during the measurement. *pyXPCSviewer* can compute simple diagnostics to aid in identifying instabilities in the sample environment or measurement conditions. In Fig. 3 ▸(*b*), we plot the averaged photon counts on the detector as a function of frame index (or time) that can be used to identify frame drops that could arise due to detector malfunction or changes in the incident beam. Users can also choose a region to zoom in to explore the fine structure, as shown in Fig. 3 ▸(*d*). The Fourier transform of Fig. 3 ▸(*b*) is shown in Fig. 3 ▸(*c*) and this can help with identifying possible vibration modes in either the beam intensity or the sample setup.

### 2.4. Multi-tau correlation visualization and modeling

Multi-tau correlation approximates *g*_2_ and characterizes the dynamics of materials in equilibrium. As the structure of a material fluctuates, the speckles fluctuate correspondingly. Components of different length scales exhibit different fluctuations. While each pixel on the area detector contains information about a certain length scale defined by the scattering geometry, a pixel usually does not have enough photon statistics to compute a high-fidelity correlation function. In practice, a *Q*-map is usually applied that groups the equivalent *Q*-regions to improve statistics while maintaining reciprocal space resolution.

**Figure 4:**
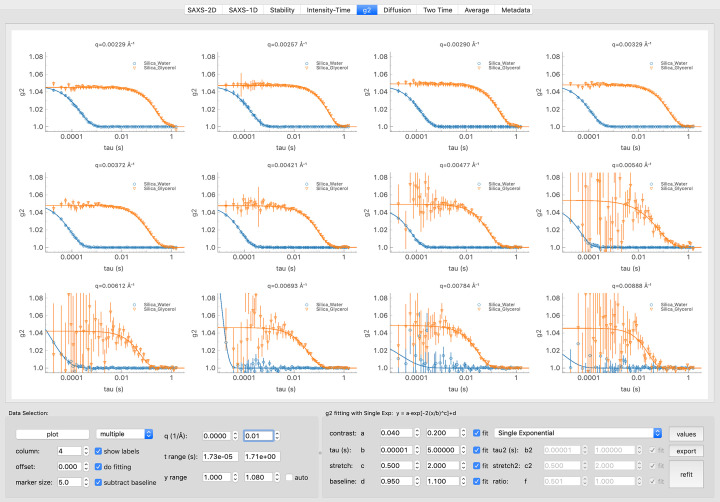
Multi-tau visualization and modeling. Users can customize the plot ranges, offset and the layout. Two widely used fitting models are included. Additional details about this module can be found in the text.

In *pyXPCSviewer*’s *g*_2_ tab that is shown in Fig. 4 ▸, users can set a *Q* range to exclude the *g*_2_ curves with large error bars. To better present the data, *pyXPCSviewer* provides three *g*_2_ display modes: (i) multiple, (ii) single and (iii) combined. In multiple mode, each *Q* value has subplots and *g*_2_ curves from different datasets are plotted in the same subplot. This mode is useful for comparing different datasets that are often the same material under different conditions. In single mode, the *g*_2_ curves of one dataset are plotted together in one image that makes it possible to compare *g*_2_ from different *Q* values. In combined mode, there is only one figure and it shows all *g*_2_ curves from all datasets.

*pyXPCSviewer* provides two widely used functions for parameterizing the decay of correlations. The single exponential decay model captures the de-correlation of simple diffusion systems represented by 

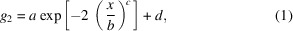

in which *a*, *b*, *c* and *d* correspond to the contrast, the characteristic decorrelation time, the stretch (compression) ratio and the baseline, respectively. For material systems that possess two-stage diffusion phenomena, the double exponential decay model is represented by 

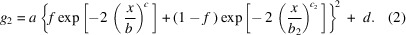

This model has a second exponential term with its decay time (*b*_2_) and stretch (compression) ratio (*c*_2_). Parameter *f* describes the fractional ratios of the two terms.

Users can define the bounds for each fitting variable. In addition, some variables can be fixed to values pre-determined at the beamline. For example, the contrast value can be calibrated with a standard static sample and thus be fixed. *pyXPCSviewer* will use the fixed values and compute the fitting uncertainty accordingly. This reduces the degrees of freedom and makes the fitting converge faster. This tab also supports the use of additional models from the user community. If the single exponential model is selected to fit the data, it is also possible to perform power-law fitting in the diffusion tab.

### 2.5. Two-time visualization

When a sample is not in a stationary state, the dynamics will not be time-invariant and so the decorrelation time-scale is a function of time. In this case, the multi-tau correlation, which assumes time-invariance, does not represent the dynamical behavior and a two-time correlation is required (Sutton *et al.*, 2003 ▸). As the name suggests, the two-time correlation *C*2(*t*_1_, *t*_2_, *Q*) computes the correlation at time *t*_1_ and *t*_2_ for equivalent *Q*.

**Figure 5:**
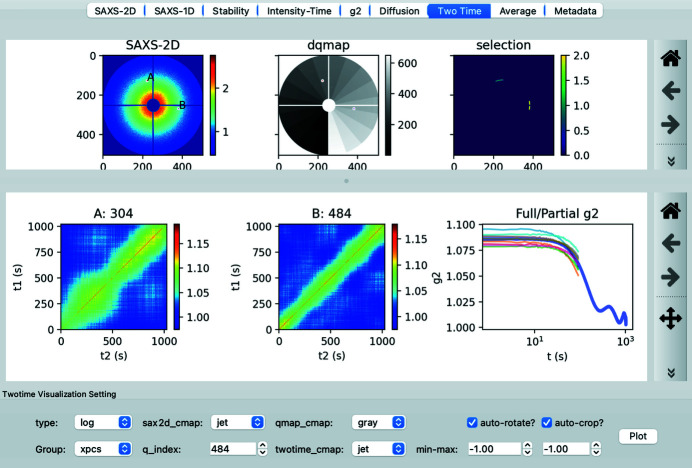
Two-time correlation visualization. The upper panels shows the scattering pattern as well as the dynamical *Q*-map that allows users to select regions (*A* and *B*) of interest to explore. The lower panels show the two-time maps for the selected points. The full (blue) and partial (red) *g*_2_ curves for selection *B* are also displayed.

To visualize the result of a two-time correlation, *pyXPCSviewer* provides two panels that are shown in Fig. 5 ▸. The upper panel plots the scattering pattern and the *Q*-map applied when computing the two-time correlation. The two plots are linked so they can be zoomed in and out simultaneously. Users can interactively select regions on either the scattering or *Q*-map plot. The two-time correlation for the last two selected points is then plotted in the bottom panel. Users can compare two correlation figures side by side. From the two-time correlation map, the *g*_2_ curves can be computed. Full *g*_2_ is obtained by averaging the whole *C*_2_ map while partial *g*_2_ curves are computed from the sub-regions. This feature can help users determine whether the sample dynamics are stationary or not.

### 2.6. Averaging of XPCS results

XPCS can provide meaningful information from samples with extremely low scattering intensities (Sheyfer *et al.*, 2022 ▸; Lurio *et al.*, 2021 ▸; Partain *et al.*, 2021 ▸), but doing so requires averaging of XPCS results from hundreds or sometimes even thousands of repeating measurements to achieve the signal-to-noise ratio (SNR) of *g*_2_ sufficient for quantitative analysis (Zhang *et al.*, 2021 ▸). To avoid prolonged X-ray exposure and mitigate the beam damage, such repeated measurements are usually performed on different locations of the sample, which makes the averaging of the results susceptible to spatial heterogeneity of the sample. These ‘outlier’ measurements can be difficult to identify manually especially when there are thousands of repeating measurements from a single sample condition. The outliers usually have *g*_2_ with a numerical value much higher than meaningful measurements due to near-zero or abnormal scattering intensity distributions and will significantly skew the statistics if not removed from the averaging.

**Figure 6:**
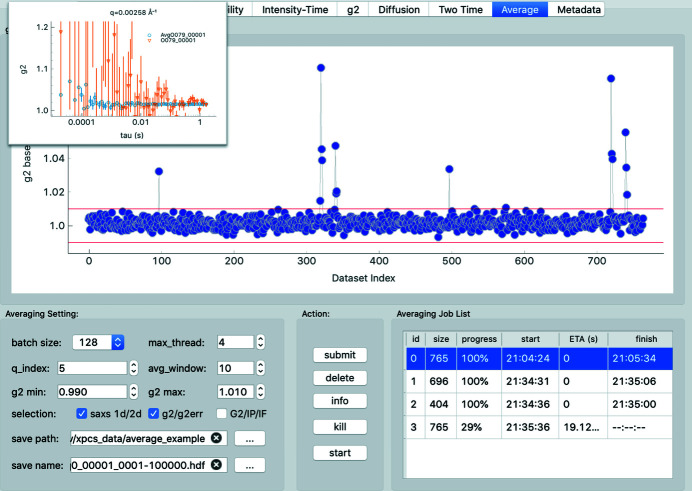
The averaging toolbox. Users set up a cutoff to remove outliers. The averaging jobs can be managed in the Action panel. The plot inset (top left) shows the *g*_2_ curves for a single dataset (orange) and the averaged result of 765 datasets (blue), showing a significant improvement in signal-to-noise ratio obtained by suitable averaging.

*pyXPCSviewer* implements an averaging toolbox in the Average tab for these purposes as shown in Fig. 6 ▸. It identifies outliers based on the *g*_2_ baseline, which is very sensitive to abnomalities in the intensity distribution within the region of interest where *g*_2_ is calculated (Sheyfer *et al.*, 2020 ▸). As shown in Fig. 6 ▸, multiple outliers can be removed by adjusting the upper and lower thresholds (blue dots that fall beyond the double red lines), and the averaging yields *g*_2_ with improved statistics as shown in the inset. Averaging also drastically reduces the data size that users need to interact with accelerating scientific interpretation of XPCS results both during and after the beam time. Future development will enhance the outlier identification with established machine learning algorithms (Pedregosa *et al.*, 2011 ▸) to pick up minute sample heterogeneity from 2-D SAXS patterns or slight radiation damage trends from stability plots that will not only lead to artifact-free XPCS results but that can also be combined with real-time XPCS analysis to enable autonomously guided XPCS measurements.

### 2.7. Metadata search

The Metadata tab gives users instant access to the metadata inside *pyXPCSviewer* without reading the file using separate software. The metadata for an XPCS dataset captures all the information about the measurement, such as the X-ray energy, frame rates and exposure time, temperature, and the synchrotron ring current. In addition, parameters passed to the correlation algorithms, such as the *Q*-map and the starting and ending frames, are also recorded in the metadata. Using the Data Exchange HDF format, the metadata is stored separately and independently from the XPCS analysis results. Using the metadata one can reproduce the conditions of the measurement and re-run the correlation algorithms. Due to the hierarchical structure of HDF files, the metadata is easily organized and stored with the data type, and units if applicable. *pyXPCSviewer* implements a simple metadata viewer function that performs a breadth-first search (BFS) to retrieve the metadata fields and shows the results in a tree structure. Users can use a string filter to search and locate any metadata fields easily.

### 2.8. Script mode

One of the essential features in *XPCSGUI* and *GIXSGUI* (Jiang, 2015 ▸) is script mode, which allows GUI functionalities to be called via a command line interface (CLI) in a programming environment (*e.g.* a Matlab terminal). This feature has been impactful for the user community over the past decade as it allows much of the XPCS data reduction process to be automated. *pyXPCSviewer* also includes a script mode, where scripts created by users can import *pyXPCSviewer*’s underlying modules and use the internal attributes and methods (*e.g.* loading HDF files, averaging of *g*_2_, *g*_2_ fitting) defined in *pyXPCSviewer*. The script mode is more efficient than the GUI when processing large batches of data. All script-mode functions, including the syntax and the mathematical formulas, are documented on the GitHub Wiki page of *pyXPCSviewer*.

## 3. Discussion and outlook

Built upon the design of *XPCSGUI*, which has been deployed and tested by users at 8-ID-I for more than a decade, *pyXPCSviewer* provides both the interactive features that allow for rapid result visualization and decision making during the beam time and script mode that streamlines data reading, fitting and plotting after beam time. Compared with the Matlab-based predecessor, *pyXPCSviewer* is open source and built with established packages and design patterns for easy maintenance and customization from the user community, a timely and essential feature given the emerging new XPCS techniques enabled by the commissioning of diffraction-limited storage rings around the world. With established Python platforms for beamline control (Bluesky) and automated data transfer (DM Workflow), *pyXPCSviewer* fits naturally into the ongoing development of a beamline and analysis scientific Python ecosystem that is being leveraged by synchrotron facilities world-wide for enhanced information exchange and more efficient use of computing resources. In addition, by enabling feedback loops between XPCS result generation and beamline control using the Python ecosystem, AI-driven autonomous scientific studies are possible by using the abundant tools for AI/ML in Python. These developments will greatly accelerate scientific discoveries in XPCS that require large-scale combinatorial studies where the data processing bandwidth of humans is a key limiting factor.
